# Liquid Profiling for Cancer Patient Stratification in Precision Medicine—Current Status and Challenges for Successful Implementation in Standard Care

**DOI:** 10.3390/diagnostics12030748

**Published:** 2022-03-19

**Authors:** Verena Haselmann, Maren Hedtke, Michael Neumaier

**Affiliations:** Institute of Clinical Chemistry, University Medicine Mannheim, Medical Faculty Mannheim, University of Heidelberg, 68167 Mannheim, Germany; maren.hedtke@umm.de (M.H.); michael.neumaier@medma.uni-heidelberg.de (M.N.)

**Keywords:** liquid biopsy, circulating tumor DNA, cell-free DNA, cancer management, personalized medicine, standard care, liquid profiling, clinical oncology

## Abstract

Circulating tumor DNA (ctDNA), accurately described by the term liquid profiling (LP), enables real-time assessment of the tumor mutational profile as a minimally invasive test and has therefore rapidly gained traction, particular for the management of cancer patients. By LP, tumor-specific genetic alterations can be determined as part of companion diagnostics to guide selection of appropriate targeted therapeutics. Because LP facilitates longitudinal monitoring of cancer patients, it can be used to detect acquired resistant mechanisms or as a personalized biomarker for earlier detection of disease recurrence, among other applications. However, LP is not yet integrated into routine care to the extent that might be expected. This is due to the lack of harmonization and standardization of preanalytical and analytical workflows, the lack of proper quality controls, limited evidence of its clinical utility, heterogeneous study results, the uncertainty of clinicians regarding the value and appropriate indications for LP and its interpretation, and finally, the lack of reimbursement for most LP tests. In this review, the value proposition of LP for cancer patient management and treatment optimization, the current status of implementation in standard care, and the main challenges that need to be overcome are discussed in detail.

## 1. Introduction

The development of next-generation sequencing (NGS) has fundamentally changed our understanding of genetic tumor evolution, including solid neoplasms, and paved the way for new treatment options enabling personalized cancer medicine, known as precision medicine. Because solid tumors are characterized by an abundance of genomic variations [1], a large number of small molecules or therapeutics that precisely target specific molecular targets that are altered in tumor cells, but not in healthy cells, have been approved for cancer treatment or are currently under investigation in clinical trials. An overview of these molecular druggable targets and their respective targeted therapeutics is provided in the OncoKB knowledge base [2]. To date, the gold standard for stratifying patients based on above molecular genetic alterations remains tissue biopsy [3]. However, tissue-based testing requires a biopsy that is associated with a general risk of complications [4,5], is unavailable in up to 30% of patients [6], and cannot be obtained frequently. Tissue-based testing is often based on archived formalin-fixed paraffin-embedded (FFPE) primary tumor tissue, which bears the risk of altered DNA and DNA cross-linking due to chemical modifications during the archiving process [7,8]. Most importantly, solid tumors exhibit spatial heterogeneity within primary site and metastases, and genetic tumor profile changes over time, particularly under the selection pressure of targeted therapy [9]. Thus, testing of tissue biopsy may fail to detect genetic alterations and does not allow the monitoring of genetic tumor evolution longitudinally.

The concept of liquid biopsy emerged almost a decade ago as an attractive alternative and represents one of the most active research areas in oncology [1]. Although liquid biopsy comprises analysis of circulating tumor DNA (ctDNA), circulating tumor cells (CTC), exosomes, and tumor-derived platelets, among others, most commonly used in precision medicine is ctDNA because it promises to negate the limitations of tissue-based genetic testing. ctDNA analysis can precisely be described by the term liquid profiling (LP) as it relies on the detection of tumor-associated genetic or epigenetic alterations in different body fluids of tumor patients. It allows researchers to assess the cumulative genetic tumor profile longitudinally in a minimally invasive manner, in real-time, from a single blood draw, as the most frequently used sample material [10]. Thus, LP mirrors intra- and intertumoral heterogeneity and, if performed over time, facilitates the detection of acquired drug-resistance mechanisms [11,12,13]. However, little is known about the origin and biological function of ctDNA, and it often represents only a minute fraction of total cell free DNA (cfDNA), which predominantly originates from hematopoietic cells [14]. The fraction of ctDNA of total cfDNA varies from 0.01% to more than 60%, depending on tumor stage, tumor type, and tumor burden, as well as treatment regime and timing of sampling [15,16,17]. Moreover, physiological and pathophysiological conditions associated with increased turnover of normal tissue or blood cells (e.g., exercise, inflammation, trauma, obesity) may alter ctDNA fraction. In addition to the low abundance, the short half-life of 15 min to 2.5 h with clearance by kidney, liver, and nuclease activity compromises diagnostic testing, as does the highly fragmented nature [12]. The mean fragment size of cfDNA of 167 bp and multiples thereof corresponds to nucleosomes [18] and suggests the predominant origin from apoptotic cells besides necrosis and active secretion [19,20]. Noticeably, it has been shown that ctDNA is 20 base pairs shorter than cfDNA from healthy cells that might be explained by differences in the nucleosomal patterning between malignant and hematopoietic cells and DNase activity [18,21].

The described inherent characteristics of ctDNA pose major challenges for detection and interpretation of liquid profiling test results. Thus, it is not surprising that a plethora of preanalytical and analytical workflows have been developed for the extraction, quantification, and further genetic testing of ctDNA, which has led to a lack of harmonization and standardization of LP testing to date. However, consensus and standard operating procedures are urgently needed for successful implementation in standard care. This review therefore focuses on (i) the main clinical applications of ctDNA testing and their potential utility in standard care, (ii) the status quo of LP implementation in routine diagnostics, and (iii) challenges/limitations that need to be addressed for LP to reach its full diagnostic potential in patient care in the future.

## 2. Clinical Application

**Figure 1:**
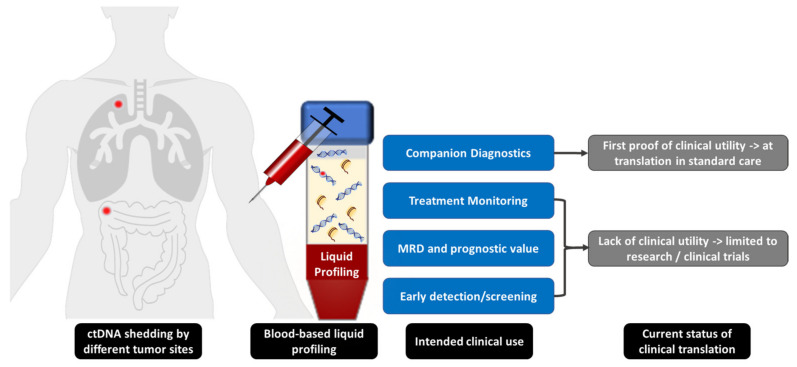
Clinical applications of liquid profiling.

The clinical value of LP by means of ctDNA analysis has been intensively investigated in numerous studies and initial clinical trials in the past decade for a wide variety of cancer types, including colorectal cancer [17,22,23], malignant melanoma [24], non-small cell lung cancer (NSCLC) [25], and breast cancer [26]. In principle, LP can be used for (i) companion diagnostics and detection of resistance mechanisms, (ii) treatment monitoring, (iii) detection of minimal residual disease (MRD) and assessment of prognostic value, and (iv) early cancer detection and screening (Figure 1). 

### 2.1. Companion Diagnostics and Detection of Resistance Mechanisms

Companion diagnostics refers to the detection of specific genetic variations as a prerequisite for the administration of targeted therapeutics. Since solid neoplasms are genetically heterogeneous, the number of druggable targets and respective tailored treatment options are continuously growing, and with it the importance of genetic tumor profiling [27]. The use of LP as alternative to tissue-based testing has been evaluated in numerous studies for different cancer types, with various levels of concordance reported between both sample materials, ranging from less than 50% to more than 90% when tissue-based testing is considered the gold standard [16,28,29,30]. Mostly, meta-analyses report a high specificity of 93.5–98.0% and a moderate overall sensitivity of 62.0% to 75.0% [31,32,33,34,35]. In principle, the level of concordance clearly depends on the testing indication, with two different scenarios. Patients with advanced disease who undergo genetic testing to select first-line targeted therapy usually have high levels of ctDNA, and the majority of variations tested are truncal mutations because they are founder mutations occurring early during carcinogenesis [3]. Such variations, like in *B-Raf proto-oncogene, serine/threonine kinase* (*BRAF)* in malignant melanoma, occur in all tumor cells and thus have a high variant allele frequency (VAF) of often >1% in cfDNA in the described setting. In these cases, a concordance level well above 90%, much higher than the overall described sensitivity of LP is reported in the literature [24,29,36,37]. The clinical utility of LP has been demonstrated for the detection of *epidermal growth factor receptor* (*EGFR)* mutations in non-small cell lung cancer (NSCLC) patients or for the detection of *KRAS proto-oncogene, GTPase (KRAS)* mutations in patients suffering from metastatic colorectal cancer (CRC) [36,38]. In NSCLC patients, comparison of patient outcomes of targeted therapy based on LP (937 patients) and tissue (5582 patients) revealed similar results (LP 13.8 month vs. tissue-based selection 10.6 month) [39]. Thus, these companion diagnostic tests are now included in national and international guidelines as alternative to tissue-based testing in cases biopsies are unavailable, of poor quality, or can only be obtained by increased risk [27]. The second scenario relates to the detection of emerging resistance mechanisms under targeted therapy. These variations are subclonal and therefore characterized by a low VAF of less than 0.1% in more than 20% of cases [17]. Common examples of acquired resistance to targeted therapy that can be detected by LP include the emergence of *KRAS* or *NRAS proto-oncogene*, *GTPase (NRAS)* mutations under anti-EGFR therapy in CRC patients [40,41] or the detection of the *EGFR* NP_005219.2:p.T790M mutation after exposure to first- or second-generation tyrosine kinase inhibitors (TKI) [42]. Other main targets include *EGFR* NP_005219.2:p.C797S or *MET proto-oncogene, receptor tyrosine kinase (MET)* amplifications as osimertinib resistance-causing variations [43], various *ALK receptor tyrosine kinase* (*ALK)* mutations in NSCLC patients under ALK inhibitors [44,45], or of *phophatidylinositol-4,5-biphosphate 3-kinase catalytic subunit alpha (PIK3CA)* or *estrogen receptor 1* (*ESR1)* variations in breast cancer patients under hormonal or endocrine treatment regimens [46,47]. Table 1 provides an overview of all molecular targets for FDA-approved drugs for solid tumors according to OncoKB.

As the clinical utility of LP has been demonstrated for companion diagnostics for certain cancer types, the Food and Drug Administration (FDA) has approved four companion diagnostic tests to date. These include the cobas EGFR Mutation Test v2 from Roche that is a quantitative PCR (qPCR)-based test to detect *EGFR* exon 19 deletions or NP_005219.2:p.L858R substitution in metastatic NSCLC patients to identify eligibility for TKI treatment as well as for *EGFR* NP_005219.2:p.T790M resistance mutation [38]. Another qPCR-based test, *therascreen* PIK3CA RGQ PCR Kit from QIAGEN GmbH, was FDA approved for *PIK3CA* mutation detection in liquid biopsy for postmenopausal, hormone receptor (HR)-positive, human epidermal growth factor receptor 2 (HER2-)-negative advanced breast cancer patients before administration of alpelisib in combination with fulvestrant [48]. Additionally, two NGS-based tests have recently achieved FDA approval: the Guardant360^®^ CDx from Guardant Health to determine *EGFR* status in NSCLC patients and the FoundationOne^®^ Liquid CDx from Foundation medicine for NSCLC, metastatic castrate resistant prostate cancer (mCRPC), ovarian and breast cancer patients before administration of TKI, PIK3CA, or poly(ADP-ribose) polymerase 1 (PARP) inhibitors [48]. An overview of all FDA-approved tests and their respective indication is provided in Table 2. However, in addition to these tests, a vast amount of research use only (RUO) assays are on the market that need to be validated as laboratory developed tests for a specific clinical indication by the respective laboratory before they can be offered in clinical care.

### 2.2. Treatment Monitoring

Monitoring of response to treatment and detection of relapse is usually based on analysis of conventional protein tumor markers and imaging. However, imaging is limited in terms of sensitivity and specificity [49] and does not allow assessment of molecular tumor evolution [50]. Since LP enables the detection of tumor associated variations in real-time, it can complement or even replace imaging in certain cases [51]. By identification of tumor-specific variations, it can be used as a personalized molecular tumor marker for surveillance of cancer patients [23,52,53,54]. The ability of LP to monitor treatment efficacy has been investigated in numerous clinical studies, and in general, ctDNA levels have been reported to correlate well with protein tumor markers and imaging findings [5,17,24,55,56,57]. Specifically, an early decrease in ctDNA levels is associated with response to therapy, whereas an increase indicates tumor progression. In some studies, LP has been found to shorten the lead-time compared to imaging by up to 10 months [24,58,59]. However, in cases of peritoneal metastases or intracranial lesions, the detectability of LP is limited, e.g., due to retention of ctDNA by the blood–brain barrier. Another limitation of LP includes the lack of topological information and the lack of standardized/optimized testing times during follow-up of patients [17,50]. Thus, imaging and laboratory findings should be evaluated integratively.

### 2.3. Minimal Residual Disease and Assessment of Prognosis

The negative prognostic value of cfDNA concentration and ctDNA positivity or level for progression-free survival (PFS) and overall survival (OS) of cancer patients has been revealed by meta-analyses [60,61]. After local therapy, analysis of ctDNA can be used for actionable health guidance by identifying MRD and thus patients at high risk of relapse [23,62,63]. This has been demonstrated in first proof-of-principle studies for several cancer types, including CRC, NSCLC, or breast cancer [11,23,58,64,65]. Importantly, this is also the case in early tumor stages. Tie et al. have shown that ctDNA positivity in stage II CRC patients indicates relapse and thus the need for adjuvant chemotherapy [23]. Conversely, negativity might indicate a complete response and thus obviate the need for adjuvant therapy. This is currently the subject of prospective clinical trials [62,63]. 

### 2.4. Early Detection/Screening

Recently, Cohen et al. were the first to report the potential value of LP for early detection of cancer when combined with conventional protein tumor markers as a pan-cancer test [66]. However, the use of this test in general population is not suitable because of the low prevalence of cancer and the resulting low positive predictive value of the test. Nevertheless, the combination of different diagnostic approaches could allow cancer screening for subpopulations of individuals at increased risk. Such an approach was reported in the DETECT-A trial, in which LP screening was limited to patients with a positive Papanicolaou test for detection of endometrium and ovarian cancer [67]. Although these study results are promising, further applications, clinical trials and large-scale prospective studies will be necessary to elucidate the feasibility and true value of LP for cancer screening. To date, there is one blood-based test, Epi proColon^®^ (Epigenomics AG, Berlin, Germany), that detects tumor-associated epigenetic changes and is FDA-approved for CRC screening [68]. Thus, the detection of epigenetic alterations could represent an attractive alternative to the detection of genetic alterations in the context of cancer screening.

## 3. Current Status of and Challenges for Clinical Implementation

The clinical utility of LP has been demonstrated for companion diagnostics, and four different kits have been approved by FDA for use in standard care. Beyond this application, the clinical validity and utility of LP has not yet been established. However, based on research and study results, LP is thought to have the potential to revolutionize diagnostics in oncology by enabling a personalized diagnostic approach through the use of individual tumor-specific biomarkers for treatment monitoring and surveillance of cancer patients, in addition to tailoring treatment to current needs arising from real-time monitoring of tumor evolution. Nevertheless, the implementation of LP in standard care remains below expectations and is progressing slowly, with few applications being integrated into routine care. Thus, there are also few studies to date reporting on the use of LP in everyday clinical practice. For example, Aggarwal et al. demonstrated that the use of plasma-based NGS testing for the routine management of stage IV NSCLC patients could identify an increased number of drug-responsive targets, allowing for improved molecularly guided therapy [5]. Soria-Comes et al. evaluated the comparability of tissue-based and blood-based genetic testing in a real world setting for NSCLC patients and reported an overall agreement of 87.4% for *EGFR* [69]. An even higher concordance of 91.7% for assessment of *KRAS/NRAS* and *BRAF* mutational status in CRC patients, as part of routine care, was recently reported by Hedtke et al. [17]. In addition, for advanced NSCLC patients, a positive impact on the clinical decision-making process and the treatment outcome was reported when using plasma-based NGS genotyping for therapeutic decisions in a real-world setting [6]. 

Despite these initial promising reports, there is consensus that several key obstacles must be overcome for the successful introduction of LP into standard care. These include technical issues such as harmonization and standardization of preanalytical and analytical workflows, quality assurance of LP testing, and comparability of interpretation and reporting of LP test results as a prerequisite for reliable diagnostics [70,71]. This is necessary to gain the confidence of physicians and patients, integrate LP into guidelines and clinical workflows, and ultimately obtain reimbursement [72]. In the following, these obstacles will be discussed in detail.

### 3.1. Technical Challenges

Technical challenges of LP result from (i) the low concentration of cfDNA, (ii) its highly fragmented nature, (iii) the low fraction of ctDNA in total cfDNA, and (iv) the background of cfDNA released from hematopoietic and healthy cells, with the risk of harboring age-related variations or alterations due to clonal hematopoiesis of indeterminate potential (CHIP) [73,74,75]. These issues must be considered in the preanalytical workflow and in the selection of an appropriate analytical method, each step of which may interfere with or bias LP assay results. 

The preanalytical workflow includes all steps from venipuncture to cfDNA extraction. Although there are no standard operation procedures to date, some general recommendations are given in guidelines such as that from the European Committee for Standardization (CEN) and its specific Technical Committee 140 for in vitro diagnostic medical devices (CEN/TC 140) [76] or by various professional societies [27,77,78]. Regarding the choice of the blood collection tubes (BCT), serum is not recommended due to the induced leukocyte lysis during the clotting process and consequent release of high molecular weight (HMW) DNA, leading to dilution of the ctDNA fraction [10,19,79]. Instead, blood should be drawn in ethylenediaminetetraacetic acid (EDTA) tubes if processed within 4–6 h [79,80] or within 24 h if stored at 4 °C [81,82]. For longer processing times of up to 72 h or even longer, the use of BCT containing cell-stabilizing agents that inhibit leukocyte lysis is recommended [81,82,83,84]. Importantly, storage temperatures below 4 °C or above 40 °C should be avoided [81,83]. The best characterized tubes include Cell-Free DNA BCT^®^ tubes (Streck, La Vista, NE, United States), Cell-Free DNA Collection tubes (Roche Diagnostics, Basel, Switzerland), and PAXgene Blood ccfDNA tubes (PreAnalytiX, Hombrechtikon, Switzerland), with no significant differences reported in terms of cfDNA yields. However, since 2017, other dedicated BCTs have been launched, but have not been systematically evaluated so far. For cfDNA isolation from plasma, blood should be processed in two consecutive centrifugation steps or by one centrifugation followed by filtration [79,83,85]. Slow centrifugation at 1600× *g* to separate plasma followed by high-speed centrifugation at 16,000× *g* to remove cell debris is usually recommended [79,83], although no effects of centrifugation force or temperature on cfDNA yield has been reported [86,87,88]. Plasma should be stored at −20 °C or below, although there is no consensus on long-term storage [79,89]. In any case, repeated freeze–thaw cycles compromise the integrity of cfDNA and should be avoided [79]. Because cfDNA is highly fragmented, with ctDNA having an even smaller fragment size, the choice of an appropriate extraction procedure is critical. Specific cfDNA isolation kits have been developed that preferentially extract small fragments, either based on spin columns, magnetic beads, or polymers [3]. Importantly, cfDNA yields and fragment size/integrity vary substantially between different kits [78,90,91], which affects ctDNA assay results. The highest cfDNA yield and lowest variability are reported for the QIAamp circulating nucleic acid kit (QIAGEN, Hilden, Germany), which is considered the gold standard [78,90,92]. The cfDNA yield can be positively influenced by a lysis step/proteinase K digestion releasing nucleic acids bound to proteins or entrapped in vesicles [70,93,94], and the elution volume or repetitive elutions [70,93]. Noteworthy, some kits have been reported to be inappropriate for certain downstream analytical procedures [94]. Finally, the method used to quantify isolated cfDNA may affect LP results. Spectrophotometric measurement is considered unsuitable for cfDNA quantification [88,95], whereas fluorimetric approaches by Qubit (Thermo Fisher Scientific, Darmstadt, Germany) have shown good correlation with absolute quantification by digital droplet PCR (ddPCR) [88]. Nevertheless, the variability of cfDNA quantification by Qubit is higher than that of ddPCR or qPCR [78], with overestimation observed for qPCR depending on the target gene [90,95]. qPCR of different sized amplicons, Bioanalyzer (Agilent Technologies Deutschland GmbH, Waldbronn, Germany), or TapeStation (Agilent Technologies Deutschland GmbH, Waldbronn, Germany), allow estimation of cfDNA integrity and thus assessment of contamination with HMW DNA. 

The choice of an appropriate, highly sensitive analytical method is of paramount importance for ctDNA analysis to detect ctDNA fractions as low as 0.01% [15]. Because the analytical sensitivity of standard molecular genetic techniques is limited to a VAF of 1–10%, a variety of different methods for LP have been developed and are currently in use. PCR-based approaches such as qPCR, co-amplification at lower denaturation temperature-based PCR (COLD-PCR) or amplification-refractory mutation system (ARMS)-PCR allow detection of known sequence variations but are often limited by their analytical sensitivity. For example, qPCR-based methods—even as an FDA-approved LP test—are only validated for a VAF > 1% and did not reach the required VAF threshold < 1% when evaluated in comparison studies [96]. Digital approaches such as ddPCR or beads, emulsification, amplification and magnetics (BEAMing) are also locus-specific, can be used in small multiplexing formats, but enable the highest analytical sensitivity. They are therefore considered the gold standard. NGS with an analytical sensitivity of 1% is not suitable for LP unless combined with unique molecular identifiers (UMI), so-called molecular barcoding [97]. Molecular barcoding allows amplicons to be traced back to the original template, thereby correcting for polymerase- and sequencing-induced errors. However, this has the disadvantage that the coverage must be increased in form of so-called ultra-deep sequencing [98]. In principle, NGS-based approaches rely on target amplification or hybrid capture, the latter also allowing detection of rearrangements. The advantages of NGS are obvious, as it enables the analysis of unknown sequence variations, copy number variations, small or large panels, or even the determination of the blood tumor mutational burden (bTMB) [99]. On the other hand, this is accompanied by a higher number of false-positive (due to CHIP, sequencing errors, benign tumors harboring somatic variations) and false-negative (due to the limited sensitivity, pipeline/alignment limitations) results compared to digital approaches. Thus, the use of two different detection methods targeting hot-spots or variations on which clinical decisions are based is recommended [9,100]. Confounding factors that must be considered in any case include amplicon size and input amount of cfDNA [9,78]. For example, Lampignano et al. found a higher VAF for ddPCR compared to NGS, likely due to the smaller amplicon size in case of ddPCR [78]. Weber et al. determined 8 ng cfDNA as the minimum required input for LP, with a significant increase in variability at lower input amounts [101]. It should be remembered that the limit of detection (LOD) is clearly dependent on cfDNA input. To achieve an analytical sensitivity of 0.01% at least 30,000 genome equivalents (GE) must be analyzed. Since usually no more than 10,000 GE can be isolated per mL of plasma, at least total cfDNA from 3 mL of plasma should be used for LP assays. Overall, a variety of different commercially available kits are on the market (most of them for research use only), but these have not yet been rigorously tested, and only a few direct comparison studies are available to determine consistency between different kits [101]. Recently, sequencing depth has been shown to vary significantly between different kits, as have detection rates for known sequence variations [101]. Finally, it is worth noting that concordance depends on the VAF of the sequence variation, with variations with a VAF > 1% showing a high degree of concordance, whereas increasing discordance is observed at lower VAF. 

Overall, harmonization of preanalytical and analytical protocols is required to obtain robust and reliable results, which are prerequisites for full implementation of LP in clinical care.

### 3.2. Interpretation of Results and Reporting

Data evaluation, bioinformatics pipelines used for NGS files, annotation of identified sequence variations, and interpretation of LP results in the clinical context can significantly affect recommendations for clinical decision making. The lack of harmonization also applies for bioinformatics pipelines currently used for LP that are still under further development [102]. Algorithms used for alignment and variant calling, such as MuTect, VarScan, or VarDict, have different performance for different VAFs, resulting in divergent results. For example, Weber et al. showed that MuTect2 failed to call many alterations even though they could be clearly identified by visual inspection of the respective binary alignment map (BAM) file in the Integrative Genomics Viewer (IGV) browser [101]. In addition, identification of non-tumor derived variations resulting from either germline, age-related variations from healthy cells, or CHIP is another major challenge [103]. To address this issue, genomic DNA (gDNA) from leukocytes can be sequenced in parallel with cfDNA to subtract CHIP or germline variations. However, this increases time and cost. Bioinformatics subtraction of these confounding variations is an attractive alternative [104], but increases the risk of bias. Interpretation of variations with a low VAF is generally difficult because differentiation between false-positive results and subclonal variants may not be possible. Most importantly, there is as yet no consensus on clinically relevant VAF thresholds that justify a change in treatment regimen [17,105]. On the other hand, there are also false-negative results that can be associated with response to therapy, contamination of the sample with wild-type DNA, or the lack of ctDNA shedding. It may not be possible to distinguish between true-negative results and the absence of sufficient amount of ctDNA—unless the presence of ctDNA can be confirmed by other variants, such as when NGS is used for LP [77,103]. Therefore, terms such as “not detected” should be preferred over “negative”, and tissue analysis or, if this is not feasible, re-testing over time should be recommended [77,103]. Finally, for detection of resistance mechanisms it is important to consider that resistant subclones are expected to shed less ctDNA compared with sensitive cells [51]. Consequently, LP results need to be evaluated in a diagnostic and clinical context along with imaging, further laboratory results, and clinical findings.

### 3.3. Quality Control

Quality assurance by the mean of internal and external quality control (QC) is critical to ensure reliable test results [106]. Internal QC of the preanalytical workflow should assess cfDNA yield and integrity [71]. For evaluation of ctDNA analysis, appropriate external controls should be analyzed in parallel. Unfortunately, these are included in the minority of commercially available kits. In most cases, reference material offered by companies such as SensID or SeraCare must be used as an alternative, or controls can be prepared by sonification or MNase degradation (to match the size of cfDNA) of gDNA isolated from cell lines [78,95]. Controls should have the same concentration as cfDNA from patient samples and contain variations that cover the range of naturally occurring VAFs. When using an assay to detect a known variant, at least one high VAF control, one at the LOD, and one wild-type control should be analyzed. In a multiplex setting or with large panels, it is not feasible to include a control for each locus, but at least one should be included for each type of variant and/or common hotspots. Regarding external QC, external quality assessment (EQA) schemes are available from several EQA providers, the first of which was offered by the Reference Institute for Bioanalytics (RfB) back in 2016 [95]. However, to date, there are no EQAs for bioinformatics pipelines and annotation of identified sequence variations.

### 3.4. Clinical Acceptance

The successful implementation of a new biomarker into clinical practice requires integration into guidelines and clinical workflows [107]. Therefore, clinicians must be convinced of the utility of the particular biomarker for their patients. In the case of LP, there is still disagreement among oncologists about the value and reliability of ctDNA analysis [103]. This could be due to heterogeneous study reports [32] and disappointing results from some laboratories [108], which can be explained by the use of inappropriate archived samples in numerous studies [109] and the lack of quality standards and harmonized workflows. In addition, prospective studies demonstrating the clinical utility of LP, a rapid turn-around time, the establishment of clinically relevant cut-offs that justify a change in treatment regimen, and appropriate timing of LP requests, are mandatory for successful clinical implementation [17,72]. However, a few reports describe successful translation into routine management of cancer patients [6,17].

### 3.5. Reimbursement

To date, reimbursement options are limited to a few applications in several countries [72]. For instance, for NSCLC patients, analysis of *EGFR* mutation status and detection of the emergence of resistance-causing variation NP_005219.2:p.T790M is reimbursed [110]. The same is true for other FDA- or European Medicines Agency (EMA)-approved tests in several countries. Because the lack of reimbursement is considered one of the major obstacles hampering translation into standard care, and because recognition by health insurers is a lengthy process, the urgent need for prospective large-scale clinical trials for promising LP applications becomes obvious [72]. Interestingly, the benefits of LP have been positively evaluated in initial cost-effectiveness studies, and recent increases in private and public payer reimbursement for LP testing have been noted [111].

## 4. Conclusions

In conclusion, the clinical applications of LP are as versatile as the preanalytical, analytical, and bioinformatics workflows. The lack of harmonization and standardization is considered the major challenge for successful integration in routine cancer patient care. Thorough validation of the entire workflow from venipuncture to reporting of results, use of appropriate internal quality controls and participation in EQAs are of utmost importance to ensure reliability of test results. For implementation in clinical workflows, regular exchange between the disciplines involved, e.g., within tumor boards, is mandatory. This enables an integrative evaluation of LP in the diagnostic and clinical context as a prerequisite for LP to develop its full diagnostic power. In our own experience, recognition by clinicians and integration into routine care is possible for LP-based companion diagnostics, although it requires time and patience. Most importantly, analytical test results must be reliable to gain clinicians confidence. Once accepted, further applications such as use as an individual biomarker through development of personalized assays for tumor-specific alterations could be the next achievable step. Ultimately, the next years will shed light on the true value of LP as a complementary diagnostic tool for the management of cancer patients in routine diagnostics.

## Figures and Tables

**Table 1:** Druggable targets in solid neoplasms modified according to OncoKB.

Gene	Alteration	Cancer Type
*ALK*	Fusions, Oncogenic Mutations	NSCLC
*ATM*	Oncogenic Mutations	Prostate Cancer, NOS, Prostate Cancer
*BARD1*	Oncogenic Mutations	Prostate Cancer, NOS, Prostate Cancer
*BRAF*	V600	Melanoma
*BRAF*	V600E	Anaplastic Thyroid Cancer, CRC, NSCLC
*BRAF*	V600E, V600K	Melanoma
*BRCA1*	Oncogenic Mutations	Ovary/Fallopian Tube, Ovarian Cancer, Peritoneal Serous Carcinoma, Prostate Cancer, NOS, Prostate Cancer
*BRCA2*	Oncogenic Mutations	Ovary/Fallopian Tube, Ovarian Cancer, Peritoneal Serous Carcinoma, Prostate Cancer, NOS, Prostate Cancer
*BRIP1*	Oncogenic Mutations	Prostate Cancer, NOS, Prostate Cancer
*CDK12*	Oncogenic Mutations	Prostate Cancer, NOS, Prostate Cancer
*CHEK1*	Oncogenic Mutations	Prostate Cancer, NOS, Prostate Cancer
*CHEK2*	Oncogenic Mutations	Prostate Cancer, NOS, Prostate Cancer
*EGFR*	Exon 19 deletion, L858R	NSCLC
*EGFR*	Exon 20 insertion	NSCLC
*EGFR*	G719	NSCLC
*EGFR*	L861Q	NSCLC
*EGFR*	S768I	NSCLC
*EGFR*	T790M	NSCLC
*ERBB2*	Amplification	Breast Cancer, Esophagogastric Cancer
*FANCL*	Oncogenic Mutations	Prostate Cancer, NOS, Prostate Cancer
*FGFR2*	Fusions	Bladder Cancer, Cholangiocarcinoma
*FGFR3*	Fusions	Bladder Cancer
*FGFR3*	G370C, R248C, S249C, Y373C	Bladder Cancer
*IDH1*	R132	Cholangiocarcinoma, Intrahepatic Cholangiocarcinoma
*KIT*	A502,Y503dup, K509I, N505I, S476I, S501, A502dup	Gastrointestinal Stromal Tumor
*KIT*	A829P and 5 other alterations	Gastrointestinal Stromal Tumor
*KIT*	D572A and 65 other alterations	Gastrointestinal Stromal Tumor
*KIT*	K642E	Gastrointestinal Stromal Tumor
*KIT*	T670I	Gastrointestinal Stromal Tumor
*KIT*	V654A	Gastrointestinal Stromal Tumor
*KRAS*	G12C	NSCLC
*KRAS*	Wildtype	CRC
*MET*	D1010, Exon 14 deletion, Exon 14 splice mutation	NSCLC
*NF1*	Oncogenic Mutations	Neurofibroma
*NRAS*	Wildtype	CRC
*NTRK1*	Fusions	All Solid Tumors
*NTRK2*	Fusions	All Solid Tumors
*NTRK3*	Fusions	All Solid Tumors
*PALB2*	Oncogenic Mutations	Prostate Cancer, NOS, Prostate Cancer
*PDGFB*	COL1A1-PDGFB Fusion	Dermatofibrosarcoma Protuberans
*PDGFRA*	Exon 18 in-frame deletions, Exon 18 in-frame insertions, Exon 18 missense mutations	Gastrointestinal Stromal Tumor
*PIK3CA*	C420R and 10 other alterations	Breast Cancer
*RAD51B*	Oncogenic Mutations	Prostate Cancer, NOS, Prostate Cancer
*RAD51C*	Oncogenic Mutations	Prostate Cancer, NOS, Prostate Cancer
*RAD51D*	Oncogenic Mutations	Prostate Cancer, NOS, Prostate Cancer
*RAD54L*	Oncogenic Mutations	Prostate Cancer, NOS, Prostate Cancer
*RET*	Fusions	NSCLC, Thyroid Cancer
*RET*	Oncogenic Mutations	Medullary Thyroid Cancer
*ROS1*	Fusions	Non-Small Cell Lung Cancer
*SMARCB1*	Deletion	Epithelioid Sarcoma

Abbreviation: NOS = not otherwise specified.

**Table 2:** FDA-approved LP tests.

Company	Test	Method	Indication
Roche	cobas EGFR Mutation test v2	qPCR	Detection of *EGFR* driver mutations in patients who may benefit from tyrosine kinase inhibitor (TKI) treatment
Qiagen	therascreen PIK3CA RGQ PCR Kit	qPCR	*PIK3CA* mutation detection in liquid biopsy for postmenopausal, hormone receptor (HR)-positive, human epidermal growth factor receptor 2 (*HER*2-)-negative advanced breast cancer patients
Guardant Health	Guradant360 CDx	NGS	Detection of *EGFR* and *KRAS* mutations eligible for FDA-approved treatment in patients with NSCLC
Foundation Medicine	FoundationOne Liquid CDx	NGS	Used as a companion diagnostic to identify patients (with NSCLC, prostate cancer, ovarian cancer, breast cancer) who may benefit from treatment with targeted therapies

## Data Availability

Not applicable.
